# Expression of uPA, tPA, and PAI-1 in Calcified Aortic Valves

**DOI:** 10.1155/2014/658643

**Published:** 2014-02-17

**Authors:** Najlah Kochtebane, Abdullah Mossa M. Alzahrani, Aghleb Bartegi

**Affiliations:** ^1^Laboratoire Cardiovasculaire, Hôpital Bichat, Faculté de Medecine, INSERM, Paris, France; ^2^Department of Biology, Faculty of Sciences, King Faical University, P.O. Box 400, Hofuf, Al Hasa, Saudi Arabia

## Abstract

*Purpose*. Our physiopathological assumption is that u-PA, t-PA, and PAI-1 are released by calcified aortic valves and play a role in the calcification of these valves. *Methods*. Sixty-five calcified aortic valves were collected from patients suffering from aortic stenosis. Each valve was incubated for 24 hours in culture medium. The supernatants were used to measure u-PA, t-PA, and PAI-1 concentrations; the valve calcification was evaluated using biphotonic absorptiometry. *Results*. Aortic stenosis valves expressed normal plasminogen activators concentrations and overexpressed PAI-1 (u-PA, t-PA, and PAI-1 mean concentrations were, resp., 1.69 ng/mL ± 0.80, 2.76 ng/mL ± 1.33, and 53.27 ng/mL ± 36.39). There was no correlation between u-PA and PAI-1 (*r* = 0.3) but t-PA and PAI-1 were strongly correlated with each other (*r* = 0.6). Overexpression of PAI-1 was proportional to the calcium content of the AS valves. *Conclusions*. Our results demonstrate a consistent increase of PAI-1 proportional to the calcification. The overexpression of PAI-1 may be useful as a predictive indicator in patients with aortic stenosis.

## 1. Introduction

Aortic stenosis (AS) is the most common valvular disease in many Western countries and represents a major healthcare burden [1–4]. It is associated with significant mortality and morbidity. The prevalence increases with age such that it affects about 3% of the elderly population, and it is the most common reason for valve replacement [5, 6]. The main biological features observed in these pathologic aortic valves are calcification and a considerable remodelling of the extracellular matrix [7] (ECM). The ECM remodelling is involved in various physiological and pathological [8] processes and depends on the activation of different types of proteases including plasmin, a key enzyme of the fibrinolytic system [9].

In the fibrinolytic system, plasminogen activators, tissue-type plasminogen activator (t-PA), and urokinase-type plasminogen activator (u-PA) are responsible for the conversion of the abundant extracellular zymogen plasminogen into the active proteinase plasmin, the key enzyme of fibrinolysis [10]. Circulating t-PA is mainly involved in the activation of plasminogen during circulating blood clotting in the dissolution of fibrin [10] while u-PA, which is secreted by a diversity of cells of normal and neoplastic origin, binds to its specific cellular receptor (u-PAR) [11] and generates plasmin on the cell surface, a process that promotes ECM degradation and cell migration/invasion [12].

Plasminogen activators (PAs) appear to be mainly involved in pericellular proteolysis [13, 14] and their expression has been correlated to a number of physiological and pathological processes [11, 14–19]. The plasmin-generating activity of t-PA and u-PA is under the control of PAI the physiologic inhibitors of PAs. PAI-1, PAI-2, and PAI-3 are capable of neutralizing PAs by ligand binding and complex formation [11, 20]. PAI-1 is the major physiological inhibitor of plasminogen activators [21]. In most physiologic conditions and under a variety of circumstances, the production of PAI-1 is sufficient to antagonize and overcome t-PA or u-PA, but under various pathologic conditions, the levels of circulating PAI-1 may increase [22]. Previous studies have reported increased levels of PAI-1 in several pathological conditions such as breast cancer [23], myocard infraction [24, 25], and atherosclerosis [26].

The aim of this study is, first, to determine u-PA, t-PA, and PAI-1 levels in the culture media incubated with aortic stenosis valves using different ELISA kits and in a second step to investigate whether there is any correlation between the different actors of the fibrinolytic system and then to correlate their values with the valvular calcium content.

## 2. Materials and Methods

### 2.1. Valve Collection

Sixty-five aortic stenosis valves were collected immediately after cardiac surgery from patients in Bichat-Claude Bernard Hospital in Paris (France) in accordance with French ethical laws (L 1235-2 and L 1245-2 of Public Health legislation) and as part of an ongoing prospective study (GENERAC-ClinicalTrials.gov, NCT00647088; Ethics committee agreement no. 0711662 (CPP Paris Ile-de-France 1-Hotel-Dieu)). The investigation conforms with the principles outlined in the Declaration of Helsinki.

### 2.2. Preparation of the Conditioned Media with Aortic Stenosis Valves

Valves were incubated in DMEM culture medium with antibiotics (100 U/mL penicillin, 100 ng/mL streptomycin, and 0.05 ng/mL amphotericin B) (3 mL/g tissue (wet weight)) for 24 hours at 37°C in a 5% CO_2_-95% air atmosphere. The conditioned media and the valves were then frozen at −20°C.

### 2.3. Detection of Fibrinolytic Enzymes and Inhibitors Using ELISA

Concentrations of urokinase- and tissue-plasminogen activators (u-PA, t-PA) as well as plasminogen activator inhibitor-1 (PAI-1) were measured in the valve-conditioned media, using ELISA assays according to the manufacturer's instructions (Hyphen Bio Med and Technoclone). Because of the absence of a control group, the levels of plasminogen activator and inhibitor obtained in the conditioned media of the pathological valves were compared to the normal range of each protein according to the device of ELISA used. Normal human plasma range of u-PA, t-PA, and PAI-1 is ≤5 ng/mL, ≤10 ng/mL, and ≤25 ng/mL, respectively.

### 2.4. Quantification of the Calcification of the Valves

The DEXA (“Dual-Energy X-Ray Absorptiometry”) is a noninvasive technique for determining the human or animal bodies' composition. The calcium content of each valve was measured, *ex vivo*, as the bone matrix content by this technique using a PixiMus densitometer (Lunar Corp., Madison, WI).

### 2.5. Statistical Analysis

Results are reported as means ± standard deviation. Correlation analyses between the levels of u-PA, t-PA, and PAI-1, in the conditioned media with aortic stenosis valves, were performed by calculating the coefficient of Spearman. Only Spearman Rho (*r*_*s*_) and probability (*P*) values corrected for ties are indicated. Values higher than 0.5 are considered significant.

## 3. Results

### 3.1. Characteristics of the Group

Sixty-five aortic valves were collected immediately after cardiac surgery from patients suffering from aortic stenosis. The average age of the patients operated on is 75 years; 62% of the patients are male and 38% are female. The mean BMC in the AS valves is 0.21 ± 0.13 (Table 1).

### 3.2. The Level of u-PA, t-PA, and PAI-1 in the Conditioned Media of the AS Valves

Samples of each conditioned medium with AS valves were analysed in the plasminogen activators microtiter ELISA, for u-PA and for t-PA. The u-PA and t-PA measured levels differ slightly between the patients with aortic stenosis and were ranged from 0.33 to 3.75 ng/mL and 0.84 to 6.93 ng/mL, respectively. The mean concentrations of u-PA and t-PA of the whole studied conditioned media are 1.69 ng/mL ± 0.80 and 2.76 ng/mL ± 1.33, respectively.

**Figure 1:**
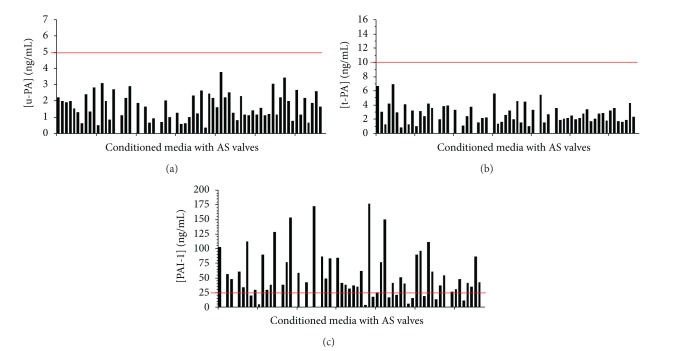
Concentrations of u-PA, t-PA, and PAI-1 in the conditioned media with aortic stenosis valves. All the aortic stenosis valves showed normal u-PA (a) and t-PA (b) concentrations, and forty-five valves showed elevated PAI-1 levels (c) when compared to the normal human plasma range according to the u-PA, t-PA, and PAI-1 microtiter ELISA device (the upper limit of which is shown as the red horizontal line at 5 ng/mL, 10 ng/mL, and 25 ng/mL, for u-PA, t-PA, and PAI-1 resp.). AS: aortic stenosis.

All the studied valves showed normal u-PA and t-PA concentrations (Figures 1(a) and 1(b)) when compared to the normal human plasma range according to the u-PA and the t-PA ELISA device (the upper limit of which is shown as the horizontal line at 5 ng/mL for the u-PA and 10 ng/mL for t-PA).

All the conditioned media with AS valves were also analyzed in the PAI-1 microtiter ELISA. The conditioned media level of PAI-1 differs significantly between the patients with aortic stenosis and was between 3.47 and 176.19 ng/mL. The mean concentration of PAI-1 in the whole studied conditioned media is 53.27 ng/mL ± 36.39.

Forty-five of the sixty-five pathological valves (69%) showed elevated PAI-1 levels (Figure 1(c)) when compared to the normal human plasma range according to the device of the PAI-1 ELISA (the upper limit of which is shown as the horizontal line at 25 ng/mL).

### 3.3. Correlations between the Levels of u-PA, t-PA, and PAI-1 in the Conditioned Media of AS Valves

**Figure 2:**
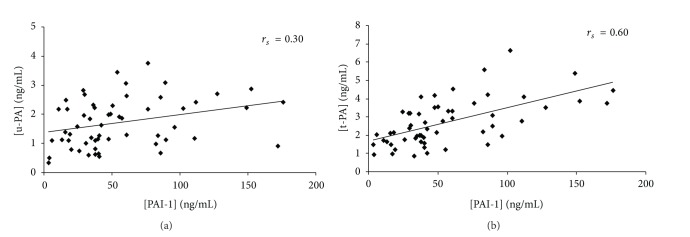
u-PA or t-PA and PAI-1 levels in conditioned media with aortic stenosis valves. Correlation between u-PA and PAI-1 measured levels (a) and the t-PA and PAI-1 measured levels (b) in the conditioned media obtained with 65 aortic stenosis valves. The search for correlation was obtained by Spearman test (*r*_*s*_).

A weak correlation was found between u-PA and PAI-1 (*r*_*s*_ = 0.30, *P* < 0.023) in conditioned media with aortic stenosis valves (Figure 2(a)), however, an important correlation was found between t-PA and PAI-1 in the same samples (*r*_*s*_ = 0.60, *P* < 0.0001) (Figure 2(b)).

### 3.4. PAI-1, u-PA, t-PA, and Calcium in the AS Valves

There was no significant difference in the average concentration of u-PA and t-PA between the different groups of the BMC valves but the mean concentration of PAI-1 increased in function of the level of calcification of the valve. For the valves with AS in which the calcium content was lower than 0.2 (g/g wet weight), the mean concentration of PAI-1 was 42.47 ng/mL ± 32 and increases to 78.75 ng/mL ± 36 when a calcium concentration is higher than 0.4 (Table 2).

## 4. Discussion

Aortic stenosis (AS) is the most frequent valvulopathy in the Western countries and is the second cause of cardiac surgery after coronary bypasses. 65 human aortic valves suffering from aortic stenosis were analysed in this study. The mean age of the operated patients with AS is 75 years, this is probably related to the slow evolution of this pathology. This result confirmed the high prevalence of the AS in the elderly. Sixty-two percent of the operated patients are male, results in accordance with previous clinical studies associated with calcific aortic valve disease which show that the male gender is associated with a twofold increased risk [27].

Valves with AS are characterised by calcification and ECM remodelling. The ECM remodelling depends on the activation of different types of proteases including plasmin, a key enzyme of the fibrinolytic system [9].

Fibrinolytic system has an inactive zymogen called plasminogen. The latter can be activated by plasminogen activators u-PA or t-PA and be converted into plasmin. Plasmin can degrade both the fibrin and the ECM directly and protect the tissues from fibrosis [11]. Among all fibrinolysis components, PAI-1 plays a central role in the pathophysiology of cardiovascular diseases. It is the major physiological inhibitor of u-PA and t-PA. The plasma PAI-1 regulates the plasmin cascade by its interaction with the t-PA or u-PA [28].

In this present study, we demonstrate that studied AS valves released enzymes of the fibrinolytic systems u-PA, t-PA, and PAI-1 in the conditioned media after 24 h of incubation. These valves expressed normal plasminogen activators concentrations but overexpressed PAI-1 (the mean concentrations of u-PA, t-PA, and PAI-1 in all the studied conditioned media were, resp., 1.69 ng/mL ± 0.80, 2.76 ng/mL ± 1.33 and 53.27 ng/mL ± 36.39). Although u-PA and PAI-1 were not correlated (*r*_*s*_ = 0.30, *P* < 0.023), t-PA and PAI-1 were strongly correlated with each other (*r*_*s*_ = 0.60, *P* < 0.0001).

A limitation of the present study was that we did not have control valves; thus the levels of plasminogen activators and inhibitor obtained in the conditioned media of pathological valves were compared to the normal range of each proteins according to the device of ELISA used.

Forty-five of the sixty-five pathological valves (69%) showed elevated PAI-1 levels. Several groups have reported excess PAI-1 in atherosclerotic plaques in humans [26, 29, 30]. These studies suggest that PAI-1 plays an important role in atherosclerosis, a cardiovascular pathology with several similarities to AS valves [31]. The increased expression of PAI-1 could inactivate the t-PA in circulation. Thus, a lower level of t-PA antigen or t-PA/PAI-1 complex and free u-PA reflect greater fibrinolytic potential and proteolytic process in the AS valves.

AS valves are characterised by an important calcification and ECM remodelling with inflammatory process [7, 32, 33]. It may be that all these perturbations are associated with higher levels of PAI-1. Additionally, in the studied enzymes of the fibrinolytic system, only PAI-1 concentration increased in function of the calcification levels of the AS valves. It has been demonstrated that this plasminogen inhibitor plays an important role in vascular calcification [34]. Besides its role in the fibrinolytic system, PAI-1 or serpin E1 plays a role in many human vascular disorders, and recent studies revealed that another serpin, serpin E2 also known as protease nexin-1 (PN-1) phylogenetically relative to PAI-1, is produced by most vascular and blood cells [35]. This serpin has a significantcontribution to the regulation of coagulation and fibrinolysis by its action on thrombin activators of plasminogen and plasmin; therefore, it can affect the vascular remodeling and the development of vascular lesions [36]. However the exact role of each of these serpins in the development of human vascular diseases is not yet clear.

Although, several studies have reported an increased level of PAI-1 in different pathological conditions [23–26], it is here for the first time that we demonstrate a consistent increase of PAI-1 levels in AS valves proportional to the rate of AS valve calcification.

## 5. Conclusion

In conclusion, our results demonstrate a consistent increase of PAI-1 content in relation to the calcification and the severity of AS valves. The overexpression of PAI-1 may be useful as an important predictive prognostic indicator in patients suffering from aortic stenosis.

## Figures and Tables

**Table 1:** Characteristics of patients (pts) and calcium content of the valves with AS. Calcium content of the valves was evaluated using biphotonic absorptiometry; results are expressed as mean ± SD.

	Total no.	Female	Male
Number of pts	65	22	43
Mean age (years)	75	80	72
Calcium content (g/g wet weight)	0.21 ± 0.13	0.16 ± 0.12	0.24 ± 0.12

**Table 2:** Description of the studied valves with AS. Calcium content of the valves and concentrations of plasminogen activators and inhibitor of the fibrinolytic system released during 24 hours by the AS valves into the incubation medium (valve-conditioned medium) were measured. Results are expressed as mean ± SD.

Number of valves	Calcium content (g/g wet weight)	u-PA (ng/mL)	t-PA (ng/mL)	PAI-1 (ng/mL)
65	0.21 ± 0.13	1.69 ± 0.81	2.77 ± 1.33	56.2 ± 41.35
31	<0.2	1.67 ± 0.82	2.98 ± 1.31	42.47 ± 32
31	0.2–0.4	1.77 ± 0.81	2.69 ± 1.39	60.43 ± 38
3	>0.4	0.99 ± 0.28	2.03 ± 0.65	78.75 ± 36

u-PA: urokinase-type plasminogen activator; t-PA: tissue-plasminogen activator; PAI-1: plasminogen activator inhibitor 1.
